# The Association Between Shift Work and Immunological Biomarkers in Nurses

**DOI:** 10.3389/fpubh.2020.00415

**Published:** 2020-09-14

**Authors:** Bjørn Bjorvatn, John Axelsson, Ståle Pallesen, Siri Waage, Øystein Vedaa, Kjersti M. Blytt, Hogne V. Buchvold, Bente E. Moen, Eirunn Thun

**Affiliations:** ^1^Department of Global Public Health and Primary Care, University of Bergen, Bergen, Norway; ^2^Norwegian Competence Center for Sleep Disorders, Haukeland University Hospital, Bergen, Norway; ^3^Stress Research Institute, Stockholm University, Stockholm, Sweden; ^4^Department of Clinical Neuroscience, Karolinska Institute, Stockholm, Sweden; ^5^Department of Psychosocial Science, University of Bergen, Bergen, Norway; ^6^Department of Health Promotion, National Institute of Public Health, Bergen, Norway; ^7^Department of Mental Health, Norwegian University of Science and Technology, Trondheim, Norway; ^8^Department of Health and Caring Sciences, Western Norway University of Applied Sciences, Bergen, Norway

**Keywords:** shift work disorder, immunity, inflammatory biomarkers, cytokines, interleukin, tumor necrosis factor-alpha, monocyte chemoattractant protein-1, blood spot method

## Abstract

**Objectives:** Shift work is associated with several negative health effects. The underlying pathophysiological mechanisms are unclear, but low-grade inflammation has been suggested to play a role. This project aimed to determine whether levels of immunological biomarkers differ depending on work schedule, self-reported sleep duration, self-reported sleep quality, and presence of shift work disorder (study 1). Furthermore, we aimed to determine whether these biomarkers differ after a night of sleep vs. at the end of a night or a day shift (study 2).

**Methods:** In study 1, 390 nurses provided blood samples after a night of sleep with the dried blood spot method. In study 2, a subset of 55 nurses also provided blood samples after a day shift and after a night shift. The following biomarkers were measured: interleukin-1alpha, interleukin-1beta, interleukin-4, interleukin-6, interleukin-8, interleukin-10, interleukin-13, monocyte chemoattractant protein-1, interferon-gamma, and tumor necrosis factor-alpha. Multiple linear regressions with adjustment for age, sex and body mass index (study 1) and ANOVAs with repeated measures (study 2) were conducted.

**Results:** In study 1, neither work schedule, number of night shifts, number of quick returns (<11 h between consecutive shifts), sleep duration, poor sleep quality, nor shift work disorder were systematically associated with most of these biomarkers. Compared with day only work, day-evening work was associated with higher levels of IL-1alpha and IL-13, quick returns were associated with higher levels of IL-1beta and MCP-1, short sleep duration (<6 h) was associated with lower levels of IL-1beta and higher levels of TNF-alpha, and long sleep duration (8+ h) was associated with higher levels of IL-13. In study 2, IL-1beta levels were higher (large effect size) both after a day shift (14% increase) and a night shift (75% increase) compared with levels after a night of sleep. Similarly, TNF-alpha levels were higher (moderate-large effect size) after a day shift (50% increase) compared to after a night of sleep. In contrast, MCP-1 levels were lower (large effect size) both after a day shift (22% decrease) and a night shift (12% decrease) compared with after a night of sleep.

**Conclusions:** We found some indications that shift work influenced immunological biomarkers. The results should be interpreted with caution due to limitations, e.g., related to the sampling procedure and to low levels of biomarkers in the blood samples.

## Introduction

Shift work is associated with a number of negative health effects, e.g., cardiovascular disorders and cancer (1, 2). Still, the underlying pathophysiological mechanisms are unclear. However, several studies suggest that low-grade inflammation may play a role. Both short sleep and sleep disturbances are associated with increased levels of pro-inflammatory biomarkers such as interleukin-1 (IL-1), interleukin-6 (IL-6) and tumor necrosis factor (TNF) (3, 4). Despite short sleep and sleep disturbances being common amongst shift workers (1), there is dearth of studies investigating the association between shift work and immunological biomarkers.

Studies investigating the link between shift work and immunity are few and show conflicting results. In a cross-sectional study among airline employees, Puttonen et al. (5) found that rotating shift work was associated with increased systemic inflammation. In a longitudinal study among 68 nurses with shift work and 28 nurses with daytime work, IL-1beta and TNF-alpha were significantly lower among shift workers at baseline, but not at 12 months follow-up. Furthermore, no effect of shift work on immunological biomarkers was present at 12 months follow-up when baseline values and job seniority were adjusted for (6). Another study among nurses showed that natural killer (NK) cell activity was reduced following night work compared to day work (7). Furthermore, a study comparing day workers with rotating shift workers showed higher levels of leucocytes in the latter group (8). The authors suggested that this reflected systemic inflammation, but since leukocyte levels vary extensively across time of day (9), it may also be attributed to group differences in circadian phase at the time of sampling. When comparing 225 shift workers with 137 day workers, no differences in IL-6, TNF-alpha, or lymphocyte count were found (10). Also, in a recent study comparing 254 night shift workers with 57 non-shift workers, no association in relation to work status was detected on a range of cytokines. However, night work was associated with increased number of monocytes and lymphocytes (11). In a clinical review, Faraut et al. (12) addressed the issue of shift work and immunity, and suggested several lines of further studies (e.g., use of non-invasive biological markers, conduct more longitudinal studies) to delineate the pathways by which circadian misalignment and short sleep may influence immunological mechanisms.

Much of the literature concerning sleep and shift work has focused on pro-inflammatory cytokines such as IL-1, IL-6, and TNF-alpha. However, the immune system serves many other functions, and there is a need to measure additional immunological biomarkers to better understand whether the interplay and complexity of immune functions are involved in how shift work may affect health outcomes. For example, there is limited knowledge about how shift work affects anti-inflammatory cytokines, such as IL-4 and IL-10, or other cytokines, such as IL-13 and interferon (IFN), which may have sleep regulatory properties (4). One complicating issue when studying healthy humans is that the blood concentrations of most cytokines are very low, and often not detectable (4).

In the present project, we collected blood from shift working nurses using the dried blood spot technique (13). This is a minimally invasive method in which blood may be collected at home or at the workplace, and by the participants themselves. This blood collection technique analyzed with quantitative antibody array technology allows for quantification of several cytokines from the same sample. By using this technique, we were able to quantify the following immunological biomarkers: IL-1alfa, IL-1beta, IL-4, IL-6, IL-8, IL-10, IL-13, monocyte chemoattractant protein (MCP)-1, IFN-gamma, and TNF-alpha.

This project consists of two studies, with two different aims. Aim of study 1: To determine whether levels of immunological biomarkers differ between nurses (*n* = 390) (a) on different shift schedules, (b) working different number of night shifts, (c) working different number of quick returns (defined as <11 h between consecutive shifts), (d) with short or long vs. normal self-reported sleep duration and (e) with good vs. poor self-reported sleep quality. Furthermore, (f) to determine whether levels of immunological biomarkers differ between nurses with and without shift work disorder. Aim of study 2: In nurses rotating between night and day work (*n* = 55), determine whether levels of immunological biomarkers differ after a night of sleep compared with levels after a night shift (both blood samples taken at about the same time) and after a day shift (blood sample taken at about 3 p.m.). Using these within-subject data, an additional aim was to study the diurnal influence on these immunological biomarkers.

## Materials and Methods

### Participants

Nurses were asked if they wanted to participate in this immunological project when completing a questionnaire in the ongoing cohort study “SUrvey of Shift work, Sleep, and Health (SUSSH)” (14). In the first immunological project (study 1), 485 nurses received the necessary equipment and instructions for sampling blood using the blood spot technique by postal mail. Only nurses who were working either day shifts only, a two-rotational schedule (day and evening shifts) or a three-rotational schedule (day, evening, and night shifts) were invited to participate. They were asked to take fasting blood samples when waking up the morning after a night of sleep, but before the start of the day shift. The participants were instructed to refrain from taking and sending samples if they had been ill or experienced fever during the last 3 days. Furthermore, nurses were asked not to take samples if they were pregnant. A total of 375 nurses returned blood samples together with a short questionnaire in which they answered questions about sleep duration and sleep quality (very good, pretty good, indifferent, pretty bad, and very bad) the night before blood sampling. Thus, response rate was 77.3%. Due to poor quality of some blood samples (insufficient magnitude; 224 samples) and missing questionnaires (4 samples), 228 nurses were sent new blood sample equipment and questionnaire. Still, not all nurses provided satisfactory blood samples, and the final number of samples with sufficient quality for analyses was 334. All nurses who participated in study 1 received a compensation of about EUR 40.

In study 2, 99 nurses working three-rotational shifts were sent blood sampling equipment. Besides the fasting blood samples taken in the morning after a night of sleep, these nurses also provided non-fasting blood samples right after the following day shift (at about 3 p.m.) and immediately after a night shift (taken at about the same time as the blood sample after a night of sleep). These nurses were also instructed not to take any samples if being ill or having fever during the last 3 days, as well as not taking samples if they were pregnant. Sixty-four nurses returned the blood samples together with a similar questionnaire as in study 1, in which the nurses answered questions about sleep duration and sleep quality the night before the first blood sample. Response rate was 64.6% in this study. Due to poor quality of some of the blood samples (27 samples) or lack of completed questionnaire (1 sample), 28 nurses were sent new blood sample equipment and questionnaire. Still, some of the samples had poor quality, resulting in data from a total of 56 participants having three samples each to be analyzed. One participant was excluded from the within-subject analyses due to sleeping during the night shift. Sample 1 from the 56 participants in study 2 was included with the samples in study 1, as all these samples were taken in the morning after a night of sleep. Each nurse in study 2 received a compensation of about EUR 100.

The most common work hours for the Norwegian nursing population are 07:00–15:00 (day shifts), 14:30–22:00 (evening shifts), and 22:00–07:30 (night shifts). Nurses working in outpatient clinics or administratively may work 08:00–16:00. Shift workers have a 35.5 h workweek, while day workers have a 37.5 h workweek.

### Blood Sampling Procedure

The dried blood spot method was used for sampling blood. This procedure is minimally invasive and considered to be easy to administer. The nurses pricked their finger with a lancet, and after removing the first blood drop, they applied a small amount of blood on four marked circles on Whatman 903 Protein Saver Snap Apart Cards. To be qualified as a good quality blood sample, the blood should fill the whole circle and should be drenched through to the back of the card. After blood collection, the filter cards were dried in room temperature for 12–24 h, and then returned to the researchers in sealed plastic bags with silica gels to prevent humidity. The filter cards were stored at −70°C until analysis.

### Blood Sample Preparation and Microarray Analyses

To elute cytokines from the dry blood samples, one circle (12.5 mm) was punched out of the blood card, minced and put into a 1.5 ml Eppendorf tube containing 150 μl 1× PBS. The Eppendorf tube was placed on gentle shaking for 4 h in room temperature. The filter paper was removed, and the sample was centrifuged 13,000 RPM for 3 min. Then 80 μl eluate was pipetted onto the antibody array slide Quantibody^®^ Human inflammation Array 1 (RayBiotech, Inc., Norcross, GA, USA) and analyzed according to the manufacturer's instructions. After analysis the antibody array slides were stored at 4°C and sent to RayBiotech Life, Inc, USA, for scanning (Innopsys Innoscan 710), data extraction and calculation of the concentration of each cytokine in pg/ml.

Quantibody^®^ uses the principle of microarray (forward phase) and enables the detection of several different proteins. In the current project, the following immunological biomarkers were measured: IL-1alpha, IL-1beta, IL-4, IL-6, IL-8, IL-10, IL-13, MCP-1, IFN-gamma, and TNF-alpha. Table 1 shows the limit of detection (LOD) (provided by RayBiotech Life) of the analyses for each immunological parameter. Most proteins, with the exception of MCP-1, had a high percentage of values below LOD. However, as the data are epidemiological, we kept all data, as suggested by Whitcomb and Schisterman (15). In cases were protein levels were not detected (=0), we substituted 0 with a random number between 0 and the lowest value detected for each specific protein. The statistical results remained the same irrespective of imputation or no imputation.

**Table 1:** Statistics of the limit of detection (LOD) for each immunological biomarker.

**Biomarker**	**LOD (pg/ml)**	**% below LOD**
IL-1alpha	9.1	73.9
IL-1beta	3.2	38.7
IL-4	3.3	78.4
IL-6	4.7	92.0
IL-8	9.7	84.1
IL-10	0.3	85.6
IL-13	1.1	85.1
MCP-1	3.6	0.2
IFN-gamma	0.8	64.6
TNF-alpha	2.3	77.4

*IL, interleukin; MCP, monocyte chemoattractant protein; IFN, interferon; TNF, tumor necrosis factor. Based on samples from 390 nurses*.

### Questionnaire

We collected data on self-reported sleep duration and sleep quality the night before blood sampling from a short questionnaire sent out together with the blood sampling equipment. Furthermore, we had data from the invitation questionnaire of the SUSSH cohort. From that questionnaire we collected information about the nurses' age, sex, marital status, work time equivalent, weight, height, work schedule, number of night shifts worked the last year, and number of quick returns worked the last year. In addition, presence of shift work disorder (SWD) was measured with three questions based on the criteria from the third edition of the International Classification of Sleep Disorders [ICSD-3; (16)]. The questions were: (a) Do you have a work schedule that sometimes overlap with the time you usually sleep?, (b) If yes, does this cause insomnia and/or excessive sleepiness due to reduced amount of sleep?, (c) If yes, has this lasted for at least 3 months? Participants were classified as having SWD if they responded “yes” to all three questions. Body mass index was calculated as weight (kg) divided by the square of height (meters).

### Statistics

The statistical analyses were conducted with IBM SPSS Statistics 25 for Windows. Due to positive skewness, all statistical analyses were conducted with log-transformed cytokine values. In study 1, multiple linear regressions were conducted with the different immunological biomarkers as dependent variables, and with adjustment for age, sex, and body mass index [since these variables are shown to influence cytokine levels (17)]. Predictors were work schedule [day only (reference), two-shift rotation, three-shift rotation], number of night shifts during the last year [0 (reference), 1–30, >30], number of quick returns during the last year [0 (reference), 1–30, >30], sleep duration [<6 h, 6–7.9 h (reference), 8+ h], sleep quality [very good/pretty good (reference), indifferent/pretty bad/very bad], and shift work disorder [no (reference), yes]. In study 2, one-way ANOVAs for repeated measures (Wilks' Lambda) with *post-hoc* LSD tests and effect sizes (multivariate partial eta squared in which 0.01, 0.06, and 0.14 suggest small, moderate, and large effect sizes, respectively) were used to compare intra-individual values of the different immunological biomarkers after a night of sleep, after a day shift, and after a night shift. Significance level was set to 0.05.

### Ethics

The study was approved by the Regional Committee for Medical and Health Research Ethics of Western Norway (REK-West, no 088.08). Informed consent in written form was obtained from all participants.

## Results

Most of the immunological biomarkers, with the exception of IL-1beta and MCP-1, were present in very low amounts, and with a high percentage of values below LOD (Table 1). Histograms showing the values for the different cytokines are available as a Supplementary File.

The participants in study 1 (*n* = 390) were on average 40.5 years (SD = 8.5), and 93.6% were females. Other demographic information as well as data regarding body mass index, work schedule characteristics, number of night shifts worked the last year, number of quick returns worked the last year, self-reported sleep duration and sleep quality, and shift work disorder are provided in Table 2.

**Table 2:** Questionnaire data among shift working nurses (*n* = 390) participating in study 1.

	**Mean (SD)**
Age (missing = 0)	40.5 (8.5)
Body mass index (BMI) (missing = 2)	25.1 (4.5)
	**% (*****n*****)**
**Sex (missing** **=** **1)**	
Female	93.6 (364)
Male	6.4 (25)
**Marital status (missing** **=** **1)**	
Married/cohabiting	76.6 (298)
Single	23.4 (91)
**Work time equivalent (missing** **=** **1)**	
<50%	0.0 (0)
50–75%	15.4 (60)
76–90%	18.8 (73)
>90%	65.8 (256)
**Work schedule (missing** **=** **1)**	
Day only	27.8 (108)
Day-evening	33.9 (132)
Day-evening-night	38.3 (149)
**Number of night shifts last year (missing** **=** **1)**	
0	51.7 (201)
1–30	26.2 (102)
>30	22.1 (86)
**Number of quick returns last year (missing** **=** **5)**	
0	22.9 (88)
1–30	38.7 (149)
>30	38.4 (148)
**Sleep duration^a^** **(missing** **=** **3)**	
<6 h	25.6 (99)
6–7.9 h	62.3 (241)
8+ h	12.1 (47)
**Sleep quality^a^** **(missing** **=** **3)**	
Very good/pretty good	69.8 (270)
Indifferent/pretty bad/very bad	30.2 (117)
**Shift work disorder (missing** **=** **6)**	
Yes	33.1 (127)
No	66.9 (257)

a *The night before blood sampling*.

Table 3 presents the levels of the immunological biomarkers in relation to different groups of the shift working nurses (study 1). In relation to work schedule, day-evening work was associated with significantly higher levels of IL-1alpha and IL-13 than day only work (Table 4). In relation to number of nights worked last year, working more than 30 night shifts last year was associated with a near-significant higher MCP-1 level than not working night shifts (Table 4). In relation to number of quick returns worked last year, nurses working between 1 and 30 quick returns last year had higher IL-1beta levels, and nurses working more than 30 quick returns had higher MCP-1 levels in comparison to not working quick returns (Table 4). In relation to sleep duration the night before blood sampling, short sleep duration (<6 h) was associated with lower IL-1beta levels and higher TNF-alpha levels, in comparison to a sleep duration of 6–7.9 h (Table 4). Furthermore, long sleep duration (8+ h) was associated with higher IL-13 levels. In relation to sleep quality the night before blood sampling and in relation to shift work disorder, no association to levels of cytokines was found (Table 4).

**Table 3:** Levels of immunological biomarkers in pg/ml after a night of sleep among different groups of shift working nurses (*n* = 390).

	**IL-1alpha**	**IL-1beta**	**IL-4**	**IL-6**	**IL-8**	**IL-10**	**IL-13**	**MCP-1**	**IFN-gamma**	**TNF-alpha**
	**Median, IQR**	**Median, IQR**	**Median, IQR**	**Median, IQR**	**Median, IQR**	**Median, IQR**	**Median, IQR**	**Median, IQR**	**Median, IQR**	**Median, IQR**
**Cytokine levels**	3.95, 9.45	4.65, 8.70	0.80, 3.03	0.40, 2.54	0.75, 6.97	0.07, 0.06	0.08, 0.56	174.55, 122.08	0.10, 1.55	0.50, 2.04
**Work schedule**										
Day only	2.50, 9.02	4.15, 7.10	0.50, 2.65	0.30, 2.32	0.30, 5.83	0.07, 0.06	0.08, 0.37	171.00, 132.13	0.10, 1.26	0.30, 1.74
Day-evening	5.10, 9.15	4.55, 8.17	1.10, 3.30	0.70, 2.53	2.15, 7.71	0.08, 0.17	0.10, 0.75	164.70, 129.78	0.25, 1.65	0.65, 2.04
Day-evening-night	3.70, 10.29	4.80, 10.55	0.70, 3.18	0.20, 2.69	0.10, 7.20	0.07, 0.07	0.08, 0.46	184.70, 108.35	0.10, 1.45	0.60, 2.45
**NN last year**										
0	4.20, 9.64	4.00, 7.60	1.00, 3.04	0.50, 2.64	1.10, 6.85	0.07, 0.06	0.08, 0.66	166.00, 129.55	0.20, 1.65	0.40, 2.03
1–30	3.35, 7.43	4.55, 10.03	0.45, 2.23	0.20, 2.29	0.15, 7.98	0.07, 0.07	0.08, 0.57	176.55, 127.83	0.10, 1.06	0.40, 1.60
>30	3.85, 12.16	5.70, 10.77	0.95, 3.44	0.40, 2.62	0.30, 7.33	0.07, 0.06	0.09, 0.46	185.50, 97.28	0.10, 1.67	0.80, 2.87
**QR last year**										
0	2.60, 9.23	3.35, 7.05	0.45, 2.85	0.20, 2.62	0.09, 5.71	0.07, 0.06	0.08, 0.44	164.85, 134.70	0.10, 1.41	0.30, 2.10
1–30	4.40, 9.92	5.30, 12.15	1.00, 2.78	0.40, 2.48	0.20, 7.55	0.07, 0.06	0.09, 0.56	170.60, 114.40	0.10, 1.55	0.40, 2.55
>30	3.95, 9.83	3.85, 8.98	0.85, 3.44	0.40, 2.69	1.35, 7.89	0.07, 0.07	0.09, 0.73	179.20, 128.75	0.10, 1.45	0.70, 1.95
**Sleep duration**										
6.0– <8 h	3.90, 9.52	4.90, 9.60	0.70, 2.69	0.30, 2.49	0.40, 6.85	0.07, 0.06	0.08, 0.46	176.00, 115.55	0.10, 1.45	0.40, 1.99
<6 h	4.40, 9.55	4.00, 10.62	1.00, 3.36	0.60, 3.14	0.40, 7.55	0.07, 0.07	0.08, 0.66	170.60, 132.30	0.30, 1.65	1.10, 3.02
8+ h	3.50, 9.42	4.00, 6.50	1.10, 3.52	0.30, 2.34	1.80, 8.13	0.07, 0.06	0.20, 0.72	154.20, 141.10	0.10, 1.86	0.09, 1.36
**Sleep quality**										
Good	3.65, 9.38	4.40, 7.82	0.75, 2.93	0.30, 2.46	0.55, 7.08	0.07, 0.06	0.08, 0.56	174.85, 120.90	0.10, 1.55	0.40, 2.04
Indifferent/bad	4.40, 9.75	5.50, 11.95	0.90, 3.19	0.60, 2.84	1.80, 6.99	0.07, 0.06	0.10, 0.56	176.90, 119.10	0.20, 1.50	0.70, 2.49
**SWD**										
No	4.30, 9.47	4.80, 8.60	0.80, 2.83	0.40, 2.74	0.70, 7.60	0.07, 0.06	0.08, 0.66	174.50, 124.35	0.10, 1.55	0.50, 1.94
Yes	3.70, 10.23	4.40, 10.40	0.90, 3.74	0.40, 2.14	0.90, 6.44	0.07, 0.07	0.08, 0.46	175.50, 118.00	0.10, 1.56	0.40, 2.15

*IL, interleukin; MCP, monocyte chemoattractant protein; IFN, interferon; TNF, tumor necrosis factor; NN, night shifts; QR, quick returns; IQR, interquartile range; SWD, shift work disorder*.

**Table 4:** Results from multiple linear regression analyses with different work characteristics, sleep duration, sleep quality, and shift work disorder as predictors, with adjustment for age, sex, and body mass index.

	**IL-1alpha**	**IL-1beta**	**IL-4**	**IL-6**	**IL-8**	**IL-10**	**IL-13**	**MCP-1**	**IFN-gamma**	**TNF-alpha**
	β, *p*-value	β, *p*-value	β, *p*-value	β, *p*-value	β, *p*-value	β, *p*-value	β, *p*-value	β, *p*-value	β, *p*-value	β, *p*-value
**Work schedule**										
Day only (ref)										
Day–evening	**0.14, 0.020**	0.04, 0.479	0.11, 0.071	0.08, 0.196	0.10, 0.095	0.09, 0.154	**0.13, 0.036**	0.02, 0.746	0.07, 0.271	0.09, 0.168
Day–evening–night	0.07, 0.272	−0.01, 0.823	0.05, 0.438	−0.00, 0.946	−0.02, 0.813	0.01, 0.821	0.03, 0.625	0.08, 0.184	−0.01, 0.879	0.06, 0.345
**NN last year**										
0 (ref)										
1–30	−0.05, 0.347	0.01, 0.895	−0.04, 0.467	−0.02, 0.707	−0.05, 0.319	−0.06, 0.272	−0.07, 0.176	0.07, 0.168	−0.09, 0.104	−0.05, 0.362
>30	0.02, 0.726	−0.02, 0.687	0.01, 0.829	−0.04, 0.473	−0.04, 0.456	−0.04, 0.462	−0.03, 0.635	0.10, 0.051	−0.03, 0.635	0.05, 0.388
**QR last year**										
0 (ref)										
1–30	0.11, 0.110	**0.14, 0.034**	0.05, 0.451	0.02, 0.751	0.04, 0.543	0.03, 0.670	0.00, 0.982	0.09, 0.177	−0.00, 0.962	0.00, 0.983
>30	0.09, 0.191	0.02, 0.725	0.07, 0.264	0.04, 0.559	0.10, 0.143	0.04, 0.519	0.07, 0.282	**0.13, 0.043**	0.02, 0.787	0.02, 0.743
**Sleep duration**										
6.0– <8 h (ref)										
<6 h	0.01, 0.890	**−0.11, 0.041**	0.04, 0.430	0.03, 0.573	0.01, 0.856	0.02, 0.693	0.00, 0.987	−0.03, 0.615	0.04, 0.493	**0.11, 0.040**
8+ h	0.00, 0.940	−0.02, 0.679	0.08, 0.144	0.02, 0.702	0.07, 0.178	0.04, 0.407	**0.11, 0.030**	−0.05, 0.367	0.03, 0.638	−0.06, 0.232
**Sleep quality**										
Good (ref)										
Indifferent/bad	0.07, 0.172	0.00, 0.938	0.03, 0.507	0.02, 0.770	0.03, 0.532	0.02, 0.654	0.04, 0.429	−0.03, 0.584	0.01, 0.840	0.07, 0.165
**SWD**										
No (ref)										
Yes	0.01, 0.929	−0.04, 0.492	0.01, 0.858	−0.02, 0.652	−0.02, 0.663	−0.06, 0.287	−0.03, 0.531	−0.02, 0.655	−0.05, 0.298	−0.01, 0.790

*IL, interleukin; MCP, monocyte chemoattractant protein; IFN, interferon; TNF, tumor necrosis factor; NN, night shifts; QR, quick returns; Ref, reference group; SWD, shift work disorder; β, standardized beta. Significant findings in bold. All regressions were conducted with log-transformed cytokine values due to positive skewness*.

In study 2 (subset with *n* = 55), the participants were 39.0 years (SD = 8.1) and 94.4% were females. Table 5 presents the levels of the immunological biomarkers after a night of sleep, after a day shift, and after a night shift within the same nurses. There were significant ANOVAs for IL-1beta, MCP-1 and TNF-alpha. Partial eta squared indicated large effect sizes for IL-1beta and MCP-1, and moderate to large effect size for TNF-alpha (Table 5). *Post-hoc* tests showed that IL-1beta levels were higher after a day shift and after a night shift in comparison to levels after a night of sleep. In contrast, MCP-1 levels were lower after a day shift and after a night shift compared with after a night of sleep. For TNF-alpha, *post-hoc* tests showed that the level was higher after a day shift compared to after a night of sleep (Table 5).

**Table 5:** Levels of immunological biomarkers in pg/ml after a night of sleep (morning fasting), after a day shift (afternoon not fasting), and after a night shift (morning not fasting) among shift working nurses (*n* = 55) in a within-subject design.

	**After sleep (morning sample)**		**After day shift (afternoon sample)**		**After night shift (morning sample)**			
	**Mean**	**SD**	**Mean**	**SD**	**Mean**	**SD**	**Wilks' Lamda^a^*F*(2,53)**	**Partial Eta squared**
IL-1alpha	4.6	6.4	4.9	5.4	6.4	10.2	0.99, *p* = 0.68	0.015
IL-1beta	7.2	12.2	8.2^**^	10.0	12.6^**^	18.2	**0.83**, ***p*** **=** **0.007**	**0.172**
IL-4	1.5	2.4	1.0	1.6	1.5	2.8	0.99, *p* = 0.69	0.014
IL-6	1.1	1.8	1.1	1.8	1.1	1.7	0.97, *p* = 0.40	0.034
IL-8	4.4	7.4	4.8	7.6	4.5	8.1	0.95, *p* = 0.26	0.049
IL-10	0.3	0.3	0.2	0.2	0.2	0.3	0.99, *p* = 0.69	0.014
IL-13	0.3	0.5	0.5	0.9	0.5	1.1	0.98, *p* = 0.59	0.020
MCP-1	190.9	103.3	149.8^**^	88.2	167.5^*^	92.5	**0.86**, ***p*** **=** **0.02**	**0.144**
IFN-gamma	.6	1.0	1.0	1.5	.9	2.1	0.96, *p* = 0.32	0.042
TNF-alpha	1.0	1.5	1.5^*^	1.8	1.2	1.7	**0.88**, ***p*** **=** **0.04**	**0.118**

*IL, interleukin; MCP, monocyte chemoattractant protein; IFN, interferon; TNF, tumor necrosis factor; SD, standard deviation*.

a *Analyses were conducted with log-transformed cytokine values due to positive skewness*.

* *p < 0.05 vs. “After sleep” on post-hoc Least Significant Difference (LSD)*.

** *p < 0.01 vs. “After sleep” on post-hoc LSD*.

## Discussion

We anticipated (aim of study 1) that there would be differences in several immunological biomarkers depending on work schedule, number of night shifts and quick returns, and depending on sleep duration, sleep quality, and shift work disorder. This notion was partially supported, as levels of IL-1alpha (work schedule), IL-1beta (quick returns and sleep duration), IL-13 (work schedule and sleep duration), MCP-1 (number of night shifts and number of quick returns), and TNF-alpha (sleep duration) differed between the respective groups. However, considering the approach to analyze many biomarkers, the results may indicate that neither shift work, sleep duration nor sleep quality strongly affect immunological biomarkers.

The aim of study 2 was to explore whether the measured immunological biomarkers would differ after a night of sleep compared with after a day shift and after a night shift – in a within-subject design. IL-1beta, TNF-alpha, and MCP-1 significantly differed depending on sample occasion. *Post-hoc* tests showed higher IL-1beta and TNF-alpha levels after the shifts (for TNF-alpha only after a day shift) compared with after a night of sleep, whereas MCP-1 levels were lower after the work shifts compared to after a night of sleep. Thus, there was no clear influence on the majority of these immunological biomarkers depending on whether the blood sample was taken after sleep or after work periods. It is important to note that the blood samples taken after the day shift and after the night shift were non-fasting, as compared to the fasting blood sample taken after a night of sleep. How this may have affected the results is unclear. Also, diurnal influences may have impacted the results. However, there is poor understanding of the diurnal variation of most cytokines (2, 18, 19). Most evidence concerns IL-6 where a recent meta-analysis showed levels to be lower in the morning (20). Unfortunately, in the present study most IL-6 data were under the LOD and we could not make any clear conclusions as to whether shift workers suffered from a systemic inflammation with regard to IL-6 or whether there was an alteration seen after a night shift.

With respect to the previous literature on how shift work and sleep relate to cytokines, our findings are partially in contrast with several previous studies (5, 7, 8, 12, 21) but in agreement with others (6, 10, 11). In a systematic review and meta-analysis on the link between sleep disturbance, sleep duration and inflammation, Irwin et al. (3) found 72 studies assessing IL-6, TNF-alpha, and C-reactive protein (CRP). It was reported that TNF-alpha was not associated with neither sleep disturbances nor sleep duration, whereas IL-6 was associated with sleep disturbance (higher values) but not short sleep duration, partially lending support to the present findings. Irwin et al. (3) also reported that sleep disturbance and short sleep duration were associated with higher levels of CRP, however, that inflammatory biomarker was not measured in the present study. Furthermore, neither experimental sleep deprivation nor sleep restriction have been found to be associated with IL-6, TNF-alpha, or CRP (3). The authors concluded that sleep disturbance, but not short sleep duration, was associated with higher levels of markers of systemic inflammation (3). In another systematic review, it was concluded that acute or chronic sleep loss do not influence levels of IL-4 and IL-10 (4). In the present study, short sleep duration (<6 h) was associated with lower IL-1beta levels and higher TNF-alpha levels, whereas long sleep duration (8+ h) was associated with higher IL-13 levels. However, we found no association between the immunological biomarkers and sleep quality or shift work disorder. Our findings therefore extend current knowledge of the rather weak associations between shift work, sleep, and immune functioning.

One of the findings in study 2 was reduced levels of MCP-1 after a day shift and in the morning after a night shift compared with morning levels after a night of sleep. MCP-1 is a chemo-attractant and one of the key cytokines that regulate migration and infiltration of monocytes and macrophages into different tissues around the body. It is believed to play a role in the development of atherosclerosis and probably also in autoimmune disease (22, 23). Our finding with regard to MCP-1 using a within-subject study design is novel. Further studies are needed to clarify the importance of MCP-1 in shift work.

### Strengths and Limitations

This present project has several limitations and strengths. The blood spot method allowed the nurses to take samples in their home environment, thus facilitating blood sampling after waking up in the morning after sleep. Also, the antibody array method allowed us to analyze ten different biomarkers from the same sample, which is considered to be a major strength. Furthermore, another asset was that we were able to compare levels of immunological biomarkers at the same time points (after sleep vs. after night work), as well as compare levels at different time points (sample taken in the afternoon vs. morning samples after sleep/after night shift). While participants may have been measured at different circadian phases—due to differences between individuals in terms of diurnal type or alterations within individuals across the work schedule—the present approach should be seen as pragmatic, in order to limit influence from the circadian system when frequent samples covering the entire 24 h window could not be done. The relatively large group of nurses with acceptable response rates is also an asset. Furthermore, we believe our assessment of the exposure to a number of shift work characteristics is a strength. For example, the number of night shifts and quick returns are known to contribute to circadian disruption and are also seen as risk factors for adverse health. Another strength was that we adjusted for age, sex, and body mass index in the group comparisons, as these variables are shown to influence cytokine levels (17). A major limitation with this self-performed blood sampling procedure was that many of the initial samples were of poor quality, usually due to blood not covering the whole circle of the filter cards. Considering that these participants were nurses who are trained to handle blood, it is to be expected that such a sampling procedure may be even more difficult in other populations. Another concern relates to the low levels of immunological biomarkers in the blood samples. For all biomarkers except for MCP-1 and IL-1beta, most of the samples had levels that were below the detection limit. This is however common when measuring cytokines in circulation but is clearly a limitation with the chosen method since other methods are more sensitive with regards to detecting systemic cytokines, such as IL-6. The definition of limit of detection (LOD) is the concentration or the quantity that can be detected with reasonable certainty for a given analytical procedure (15). To avoid biasing estimates it is recommended to use all data including those below LOD in epidemiological studies like the present investigation (15). Also, it is important to note that the concentration of most cytokines in healthy individuals is very low and not always detectable even with more sensitive methods (4). The results are likely to be most robust for the IL-1beta and MCP-1 findings, since more than 61 and 99% of the values were above LOD, respectively. For the other cytokines, many samples were below the lower detection level, and the results regarding these should therefore be interpreted with caution. Another limitation with the approach to include many cytokines is the risk of conducting type-I errors. We did not apply any corrections to reduce the risk for false positive findings as such an approach would have reduced the statistical power, hence increasing the risk of making type-II errors. We believe that these estimates could give directionality for future studies, e.g., as priors in a Bayesian statistic. Still, the fact that the cytokine levels were generally low, suggests that shift work does not strongly affect immunity. Still, it should be noted that some of the group comparisons may have been influenced (e.g., comparing day only to shift worker) by “the healthy shift worker effect” (24). Also, the day only group included nurses with previous shift work experience. How and if this may have affected the results however, is unclear.

Based on previous literature and the present findings, one cannot make strong conclusions regarding how shift work affects immunity. An important step in solving this issue will be to merge data from several studies (e.g., by meta-analytical approaches), increasing the power to make more accurate estimates of possible effects.

In conclusion, the present findings showed some indications of shift workers to have higher levels of inflammatory biomarkers. However, neither work schedule, number of night shifts, number of quick returns, short sleep duration, poor sleep quality, nor having shift work disorder seemed to have a major impact on the levels of a multitude of immunological biomarkers. Yet, levels of IL-1beta and TNF-alpha were higher after a day shift, levels of IL-1beta were higher after a night shift, and levels of MCP-1 were lower both after a day shift and after a night shift, in comparison with morning levels after a night of sleep, respectively, which suggests that work may acutely impact immune function.

## Data Availability Statement

Data cannot be released into a publicly accessible repository in accordance with national guidelines and local legislation due to concerns regarding participant anonymity as raised by the Regional Committee for Medical and Health Research Ethics of Western Norway (REK-West).

## Ethics Statement

The studies involving human participants were reviewed and approved by The Regional Committee for Medical and Health Research Ethics of Western Norway (REK-West, no 088.08). The patients/participants provided their written informed consent to participate in this study.

## Author Contributions

BB: collecting the data, design of the study, data analysis, interpretation of the results, and drafting the paper. SP, SW, BM, and ET: collecting the data, design of the study, interpretation of the results, and critical review of the paper. JA, ØV, KB, and HB: design of the study, interpretation of the results, and critical review of the paper.

## Conflict of Interest

The authors declare that the research was conducted in the absence of any commercial or financial relationships that could be construed as a potential conflict of interest.
