# Supplementary figures and images for: The Association Between Shift Work and Immunological Biomarkers in Nurses
Source: Front Public Health. 2020 Sep 14;8:415. doi: 10.3389/fpubh.2020.00415 (PMC7521138; doi:10.3389/fpubh.2020.00415)

Histogram

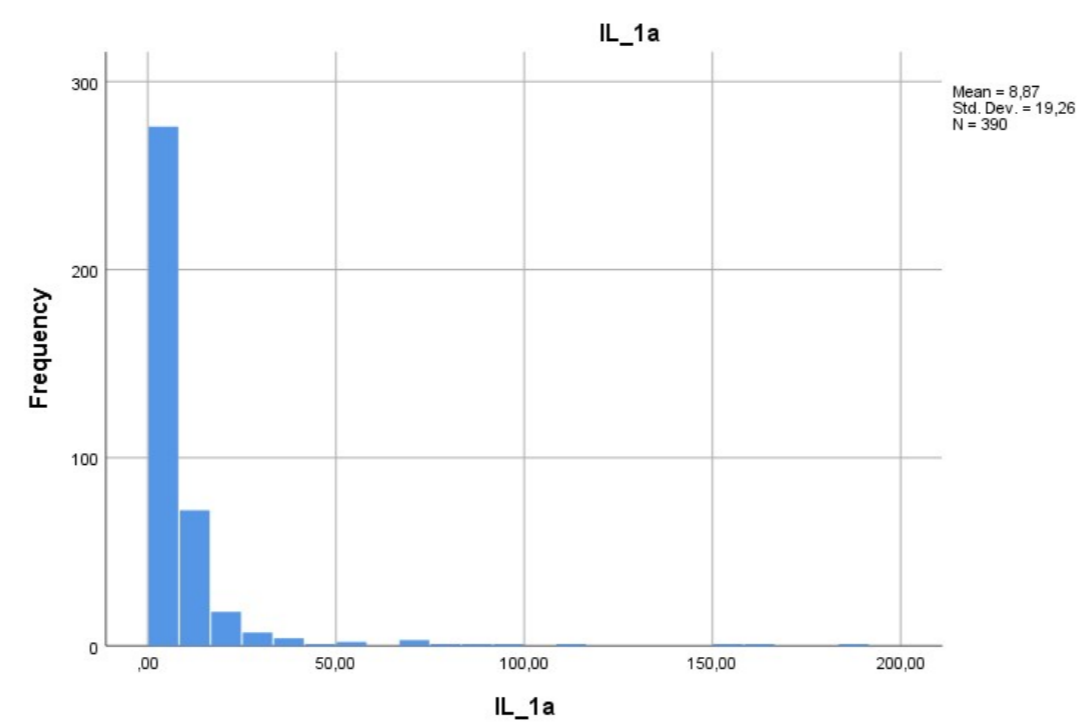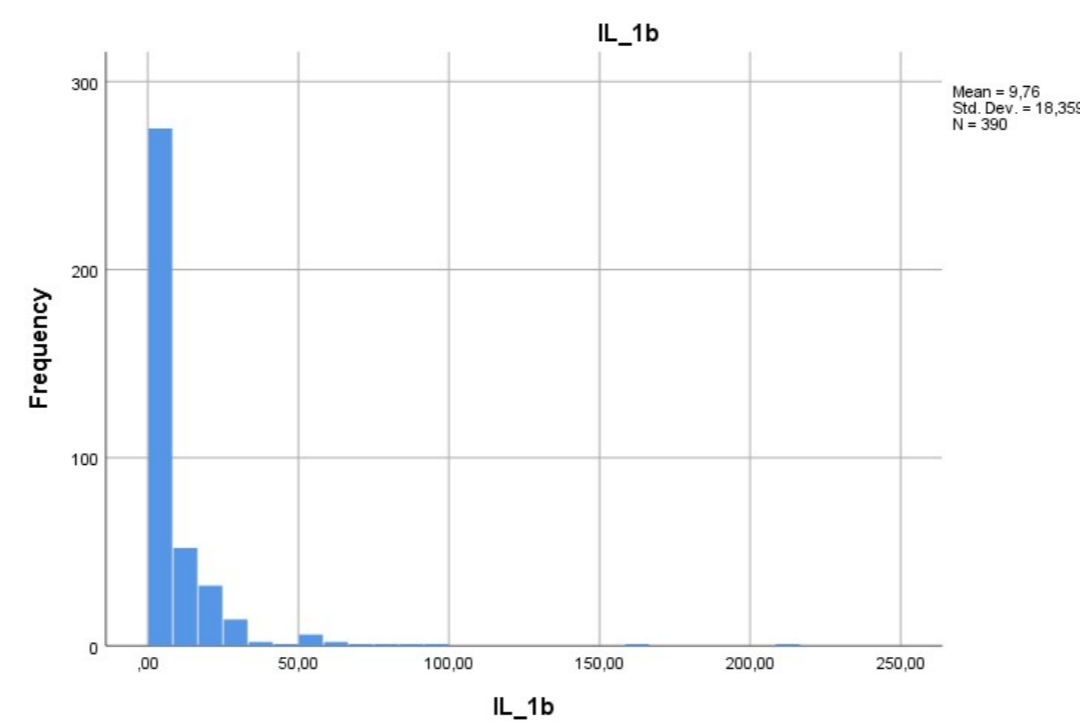

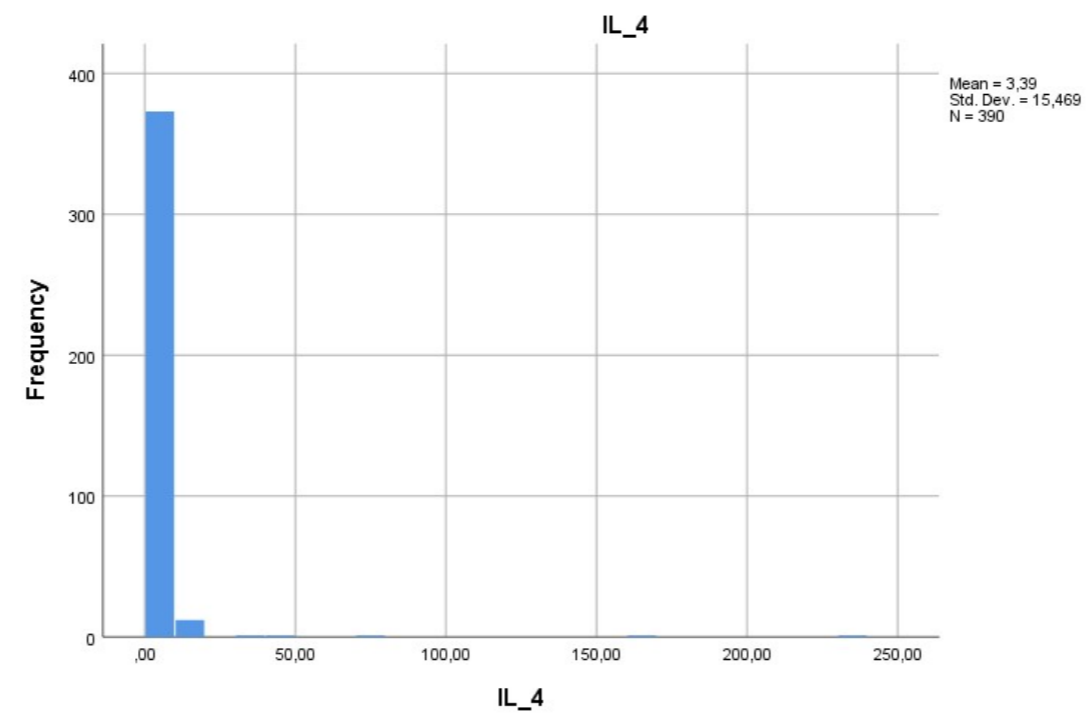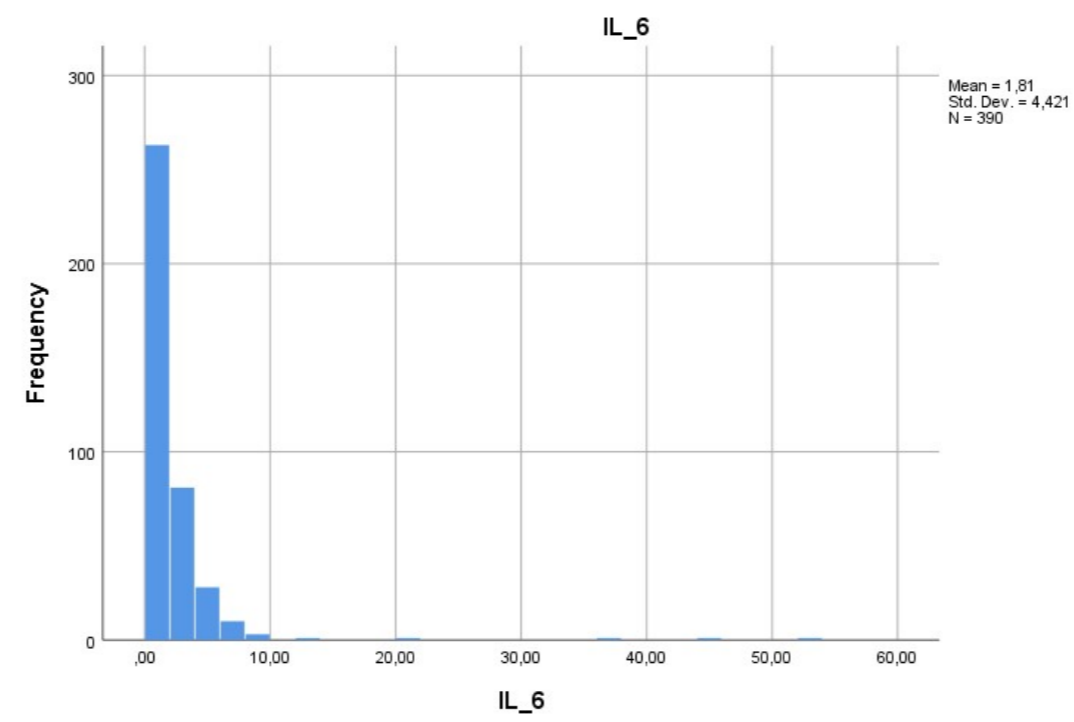

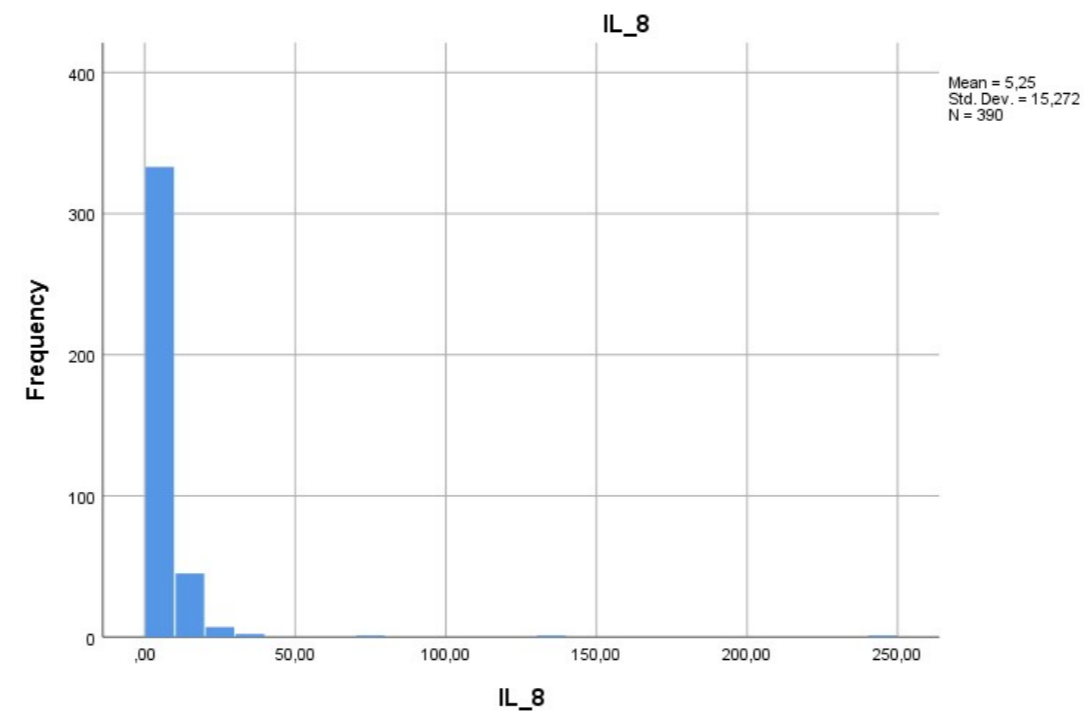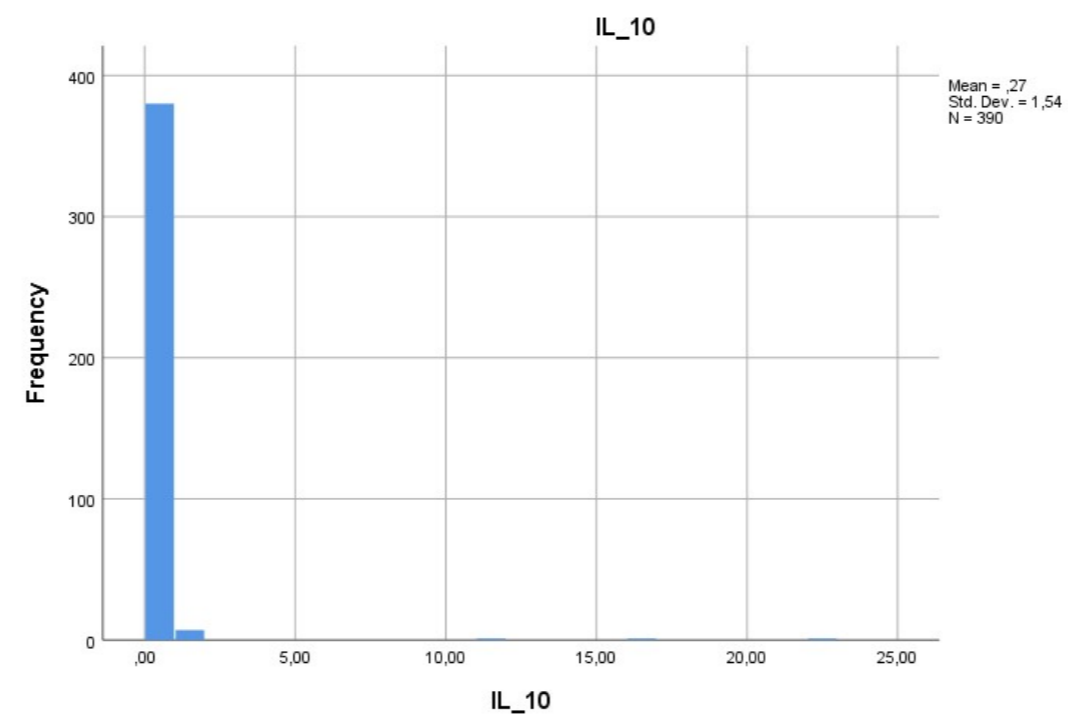

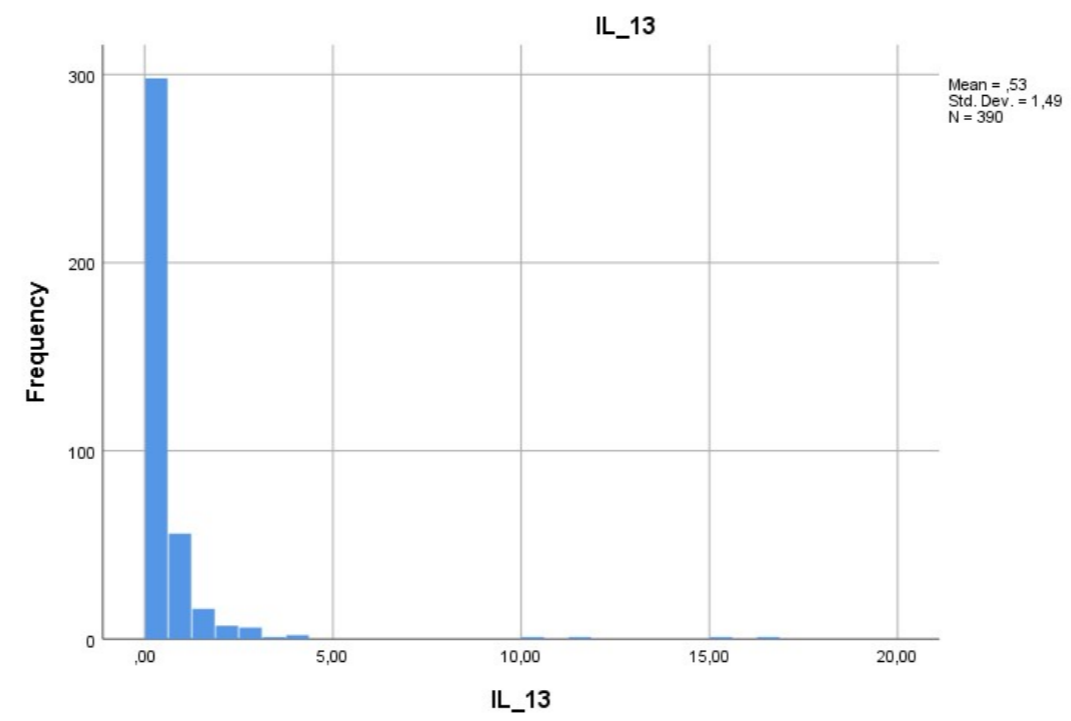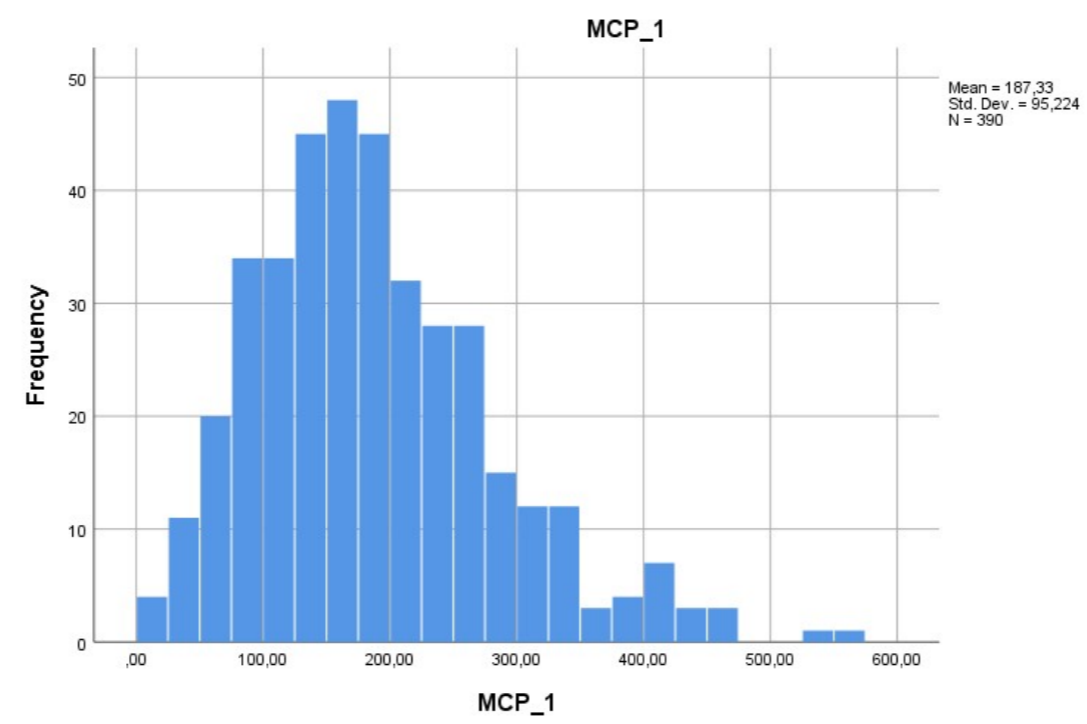

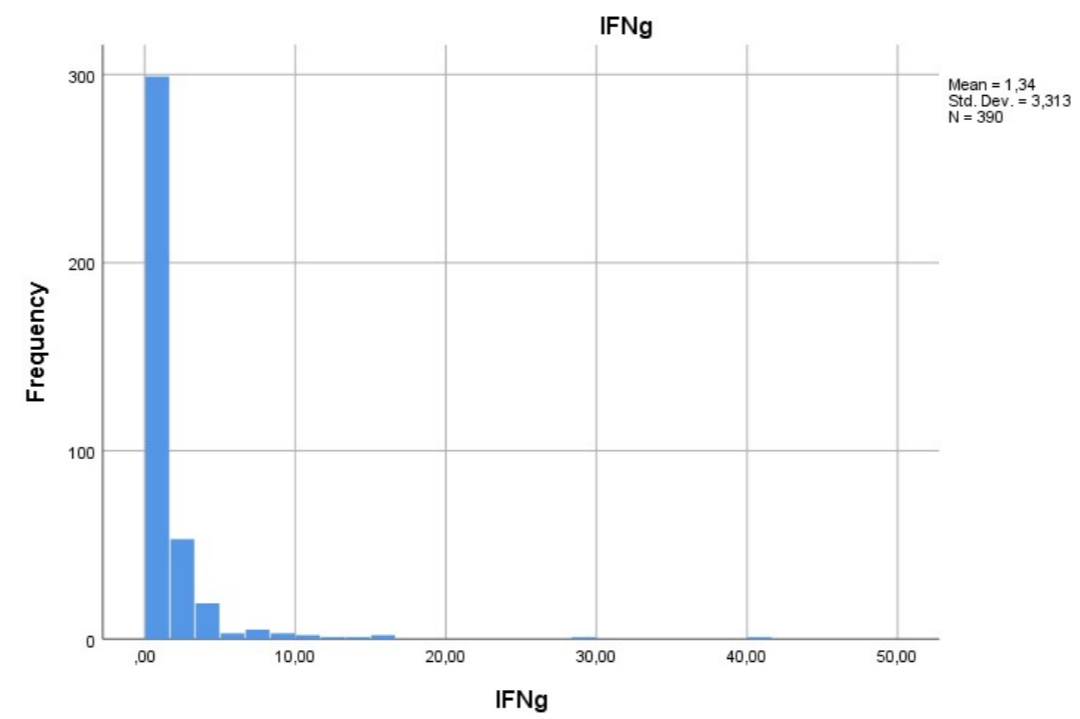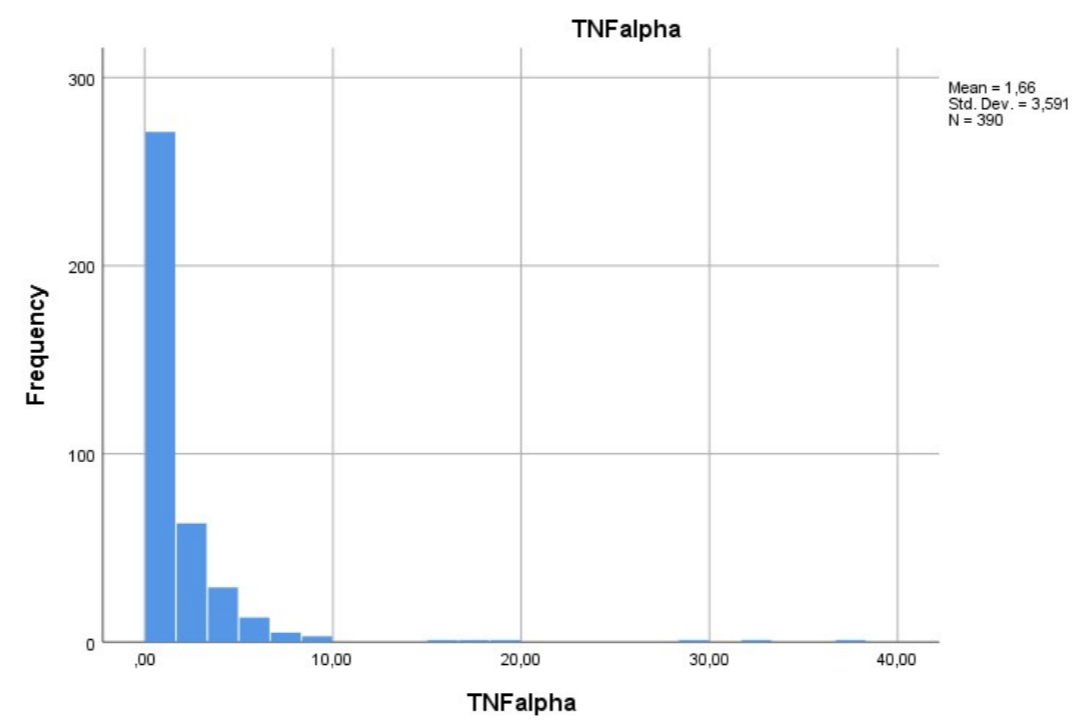

Supplement: Supplementary file 1 [file Data_Sheet_1.PDF]
